# Letter to the Editor: Innovative future concepts of extracorporeal strategies in sepsis and septic shock

**DOI:** 10.1186/s13054-023-04408-7

**Published:** 2023-03-22

**Authors:** Klaus Stahl, Christian Bode, Sascha David

**Affiliations:** 1 Department of Gastroenterology, Hepatology and Endocrinology, Hannover Medical School, Carl-Neuberg Straße 1, 30163 Hannover, Germany; 2 Department of Anaesthesiology and Intensive Care Medicine, University Hospital Bonn, Bonn, Germany; 3 Institute of Intensive Care Medicine, University Hospital Zurich, Zurich, Switzerland; 4 Department of Nephrology, Hannover Medical School, Hannover, Germany

To the Editor,

With great interest we read the recent review by Ronco et al. describing diverse technologies and concepts of extracorporeal therapy in patients with sepsis [1].

We would like to congratulate the authors for their thorough and balanced review, but also point out that all treatment modalities described here, although very different in terms of mechanism and target, have one central aspect in common, namely the *removal* of injurious factors thought to be involved in the pathophysiology of sepsis.

In fact, the negative high-quality data from both the COMPACT-2 (unselective removal using high volume coupled plasma filtration and adsorption (CPFA)) and the EUPHRATES trial (selective removal of endotoxin by polymyxin hemoperfusion) were disappointing as neither reduction in mortality nor improvement in any secondary endpoints were achieved. The COMPACT-2 trial was even prematurely determined due to a potential harmful effect of CPFA as ICU mortality was higher in the treatment group. Randomized controlled and propensity matched analyses [2] studying clinically meaningful endpoints concerning other “removal” devices are either equally disappointing, such as concerning the CytoSorb adsorber, or simply not existing as for the Seraph 100, oXiris or SepXiris adsorbers.

Considering the Hippocratic principle “Primum non nocere”, the question at least arises, whether our common dream of identifying an effective extracorporeal treatment strategy in septic shock is about to die?

Unselective adsorbing devices have the potential to remove both injurious and protective substances involved in the pathophysiology of septic organ dysfunction and compensatory attempts to maintain homeostasis. It is therefore conceivable that the unwanted extraction of protective factors may diminish beneficial physiological responses such as immune homeostasis restoration, re-establishment of endothelial integrity as well as a reduction of coagulopathy.

Therapeutic plasma exchange (TPE) is a relatively old extracorporeal strategy with a profoundly different biological approach that our group is exploring as an extracorporeal therapy in septic shock. The rationale behind TPE rather follows the idea of combining removal of excessive injurious mediators with the replacement of diminished protective factors in a singular intervention (Table 1). As such, TPE effectively removes injurious mediators such as pro-inflammatory cytokines, endothelial- and glycocalyx destabilizing factors (e.g. Angiopoietin-2, Heparanase-1) as well as molecules involved in intravascular coagulation (e.g. von Willebrand factor antigen, D-Dimers, etc.) [3–5]. At the same time, the exchange of septic plasma with that from healthy donors leads to a replenishment of protective but depleted factors involved in anti-inflammatory processes (e.g. immunoglobulins), in endothelial stabilization (e.g. Angiopoietin-1, Heparanase-2) and in anti-coagulation (e.g. Antithrombin-III, Protein C, ADAMTS-13) [3–5]. Both in a pilot prospective study [3] and in a randomized controlled trial (EXCHANGE trial) [4], additive TPE was associated with rapid haemodynamic improvement in patients with severe refractory septic shock. Of note, TPE was observed to be most effective in causing haemodynamic stabilization in patients with higher lactate concentrations at baseline suggesting an association with microcirculatory dysfunction as a potential prediction marker of TPE success in this critically ill patient cohort [4]. A multicenter randomized controlled trial (EXCHANGE-2) powered for survival is about to begin recruitment in Germany, Austria and Switzerland (NCT05726825).

**Table 1:** Molecular mechanisms targeted by additive therapeutic plasma exchange (TPE) in septic shock

Molecular mechanism targeted by TPE	REPLACE (replenished by substitution with healthy donor plasma)	REMOVE (removed by taking out patient plasma)
Immune system	Immune restauration: IgG, IgM, IgA	Pro-inflammatory cytokines, DAMPs, PAMPs
Endothelial permeability	Anti-permeability: Angpt-1, Hpa-2	Pro-permeability: Angpt-2, VEGF, sTie2, Hpa-1
Coagulation	Anti-coagulation: Protein C/S, AT-III	Pro-coagulation: vWF:Ag, D-Dimer
Microcirculation	Restoring microcirculatory function: ADAMTS-13	Causing microcirculatory dysfunction: vWF-M

Demonstrated are molecular mechanisms involved in the pathological host response of sepsis that are influenced by use of TPE. Protective, but consumed factors are replenished by TPE (REPLACE). Excessive injurious mediators are removed by TPE (REMOVE)

*Angpt-1* Angiopoietin-1, *Angpt-2* Angiopoietin-2, *ADAMTS13* A disintegrin and metalloprotease with thrombospondin-1-like domains 13, *AT-III* Antithrombin-III, *DAMPs* Damage-associated molecular patterns, *Hpa-1* Heparanase-1, *Hpa-2* Heparanase-2, *PAMPs* Pathogen-associated molecular patterns, *sTie2* A soluble receptor of tyrosine kinase with immunoglobulin-like and EGF-like domains 2, *VEGF* Vascular endothelial growth factor, *vWF:Ag* von Willebrand factor antigen, *vWF-M* (Ultra) large von Willebrand factor multimers

The late Edward Kennedy once said in a personal moment of great disappointment: *“The work goes on, the cause endures, the hope still lives, and the dream shall never die.”*

We believe that hope is not yet lost and that work on extracorporeal sepsis treatment should continue despite recent throwbacks. However, to keep the dream alive, innovative concepts should be developed—whether by combining different individual modalities, as proposed by Ronco and colleagues, or by exploring an approach aimed at rebalancing the pathophysiology of sepsis such as offered by TPE.

## Data Availability

Not applicable.
